# Comprehensive evaluation of the effects of long-term cryopreservation on peripheral blood mononuclear cells using flow cytometry

**DOI:** 10.1186/s12865-022-00505-4

**Published:** 2022-06-07

**Authors:** Bo Li, Chunmei Yang, Gui Jia, Yansheng Liu, Na Wang, Fangfang Yang, Rui Su, Yulong Shang, Ying Han

**Affiliations:** National Clinical Research Center for Digestive Diseases and Xijing Hospital of Digestive Diseases, Xijing Hospital, Air Force Military Medical University, Xi’an, 710032 China

**Keywords:** PBMCs, Cryopreservation, Adaptive immune cells, Innate immune cells, T cells

## Abstract

Human peripheral blood mononuclear cells (PBMCs) originate from hematopoietic stem cells in the bone marrow, which mainly includes lymphocytes (T cells, B cells, and natural killer cells) and monocytes. Cryopreserved PBMCs providing biobank resources are crucial for clinical application or scientific research. Here, we used flow cytometry to explore the influence of long-term cryopreservation on the quality of PBMCs with the aim of providing important evidence for the effective utilization of biobank resources. The PBMCs were isolated from the peripheral blood, which was collected from volunteers in the hospital. After long-term cryopreservation in liquid nitrogen, we analyzed the changes in cell numbers, viability, and multiple subtypes of PBMCs and studied the apoptosis, proliferation, activation, function, and status of T cells in comparison with freshly isolated PBMCs by flow cytometry, and then further tracked the effects of long-term cryopreservation on the same sample. Although the different cell types in the PBMCs dynamically changed compared with those in the freshly isolated samples, PBMC recovery and viability remained stable after long-term cryopreservation, and the number of most innate immune cells (e.g., monocytes and B cells) was significantly reduced compared to that of the freshly isolated PBMCs or long-term cryopreserved PBMCs; more importantly, the proportion of T cell subtypes, apoptosis, proliferation, and functional T cells, except for Tregs, were not affected by long-term cryopreservation. However, the proportions of activated T, naïve T, central memory T, effector T, and effector memory T cells dynamically changed after long-term cryopreservation. This article provides important evidence for the effective utilization of biobank resources. Long-term cryopreserved PBMCs can be partly used as biological resources for clinical research or basic studies, but the effect of cryopreservation on PBMCs should be considered when selecting cell samples, especially in research relating to activating or inhibiting function.

**Supplementary Information:**

The online version contains supplementary material available at 10.1186/s12865-022-00505-4.

## Introduction

Human peripheral blood mononuclear cells (PBMCs) are important biological resources that aid in improving our understanding of immunological disease and play a crucial role in immunotherapy. To address the limitations of freshly isolated PBMCs, cryopreserved PBMCs are widely used in basic studies and clinical trials. Several clinical trials necessitate the high quality of functional T cell products and stable cell subtypes; consequently, large numbers of cryopreserved PBMCs that were collected for years need to be evaluated in a suitable manner.

PBMCs are mixtures of multiple immune cells, which can be roughly divided into two immune cell subtypes, including innate immune cells (natural killer [NK] cells and monocytes) and adaptive immune cells (e.g., B cells and T cells). Furthermore, the discovery of innate lymphoid cells (ILCs) is critical for maintaining immune homeostasis or in response to inflammation [1]. T cells can be classified into CD4^+^ T (T helper cells) and CD8^+^ T (cytotoxic T cells), which can activate other immune cells and fight pathogens, respectively. Memory cells in CD4^+^ T cells respond rapidly to previously encountered antigens [2–5]. CD4^+^ T cells can also be divided into four functional subtypes: T helper (Th) 1 (interferon [IFN]-γ^+^), Th2 (interleukin [IL]-4^+^), Th17 (IL-17^+^), and regulatory T cells (Tregs), which express intracellular forkhead box P3 (FOXP3) and exhibit a strong suppressive function [6, 7]. Cytokines produced by Th1, such as IFN-α, IFN-γ, IL-2, granulocyte-macrophage colony-stimulating factor (GM-CSF), and tumor necrosis factor (TNF)-α, participate in cell immunity against pathogens and bacteria [8]. T helper 2 cell cytokines, including IL-4, IL-5, IL-6, and transforming growth factor (TGF)-β, enhance humoral immunity from allergens, bacteria, or toxins. T helper 17 cells secrete IL-17 and IL-23, which are involved in inflammatory responses or infection defense [9–11]. However, the cryopreservation process and storage time may affect the PBMC subtypes; thus, it is important to evaluate the quality of long-term cryopreserved PBMCs.

As a proven immunological assay, multi-parameter flow cytometry is widely used in cytobiology, immunology, and clinical medicine, and can rapidly and effectively analyze the diversity of PBMC subpopulations; for example, flow cytometry (FCM) can be used for disease diagnosis i.e., detecting the number of CD4^+^ T cells in the peripheral blood and monitoring the disease progression of human immunodeficiency virus (HIV) infection [12]. The human leukocyte antigen (HLA), HLA-B27, can also be detected by FCM for the diagnosis of ankylosing spondylitis [13]. Flow cytometry can also be used to measure the proportion of lymphocyte subtypes and observe the differences between healthy individuals and patients with autoimmune diseases [14].

At present, the available literature focuses on the cell viability of fresh or frozen PBMCs from different samples in biobanks [15]. Moreover, published studies have usually focused on the cellular immunological status of certain PBMC subpopulations after cryopreservation, rather than conducting a comprehensive analysis of the cell samples. Thus, in this study, we aimed to analyze the total numbers of PBMCs and the different changes in the cell subtypes at different cryopreservation time points for the same sample and to comprehensively evaluate the impact of cryopreservation on immune status by tracing the total cell number, cell viability, and markers of each subtype of immune cells in the same sample to provide important evidence for the effective utilization of biobank resources.

## Materials and methods

### Study sample preparation

From 2019 to 2020, a total of 91 blood samples were collected and tested from healthy adults. Four milliliters of peripheral blood was drawn from individuals using BD Vacutainer ethylenediaminetetraacetic acid (EDTA) tubes, which were then kept at 25 °C until the PBMCs were isolated within 4 h.

### PBMC isolation and cryopreservation

First, the peripheral blood was diluted and mixed with RPIM1640 (Gibco, Germany) at a ratio of 1:1, and then gently layered on top of 4 mL of Ficoll-Hypaque 10,771 (Sigma, USA). The tubes were centrifugated at 1600 rpm for 30 min at 25 °C. Then, the PBMC layer was aspirated and transferred into a new fresh 15 mL conical centrifuge tube. The PBMCs were mixed with RPIM1640 to wash twice at 1300 rpm for 10 min. After cell counting, fresh PBMCs were stained with different FCM antibodies and tested using a BD Canto II Flow Cytometry System. The remaining PBMCs were resuspended and cryopreserved with freezing media, including 10% dimethysulfoxide (DMSO) and 90% fetal bovine serum. The cell number was generally at least 5 × 10^6^ per tube. Lastly, the frozen PBMC samples were transported into a liquid nitrogen container for long-time preservation after being stored in the freezing container at − 80 °C overnight. 5–10 samples were processed at a time and the subsequent freez thaw process followed every step of the protocol strictly.

### Thawing the PBMCs

The cryopreserved PBMCs were placed in a 37 °C water bath for rapid thawing, and then transferred into a 15 mL conical centrifuge tube filled with pre-warmed RPIM1640. The cells were washed at 1300 rpm for 10 min, resuspended in culture medium, and incubated overnight.

### Cell proliferation assay

The carboxyfluorescein diacetate succinimidyl ester (CFSE, Biolegend, USA) was used to label the proliferation of the PBMCs. The cells were resuspended with 5 μM CFSE working fluid and incubated in a carbon dioxide cell incubator for 20 min. Next, a five-time volume of the RPIM1640 medium containing 10% fetal bovine serum was added to terminate the reaction, and then washed three times by RPIM1640 medium. After labeling with CFSE, the cells were incubated with human CD3/CD28 T cell activator (Stem Cell, Canada) at a dose of 25 µL/mL and IL-2 (Pepro Tech, USA) at a dose of 50 ng/mL for up to 3 d, and then analyzed by fluorescence-activated cell sorting (FACS).

### Cell stimulation assay

The cell suspension (1 × 10^6^) was placed into an Ultra-Low Attachment 24-well plate (Corning, USA), and 2 µL of the cell stimulating agent containing phorbol 12-myristate 13-acetate/ionomycin/Golgi body inhibitor was added to stimulate cell activation rapidly, and the plate was incubated at 37 °C in 5% CO_2_ for 4 h before the follow-up experiments.

### Inductive and supressive assays of tregs

Inductive assay: The CFSE labeled PBMCs were stimulated and induced by human CD3/CD28 T cell activator (Stem Cell, Canada) at a dose of 25 µL/mL, IL-2 (Pepro Tech, USA) at a dose of 150 ng/mL and TGF-β (Pepro Tech, USA) at a dose of 5 ng/mL for up to 3 d in 37 °C, 5% CO_2_ incubator, and then analyzed by fluorescence-activated cell sorting (FACS).

Supressive assays: The CFSE labeled PBMCs were cocultured with the Tregs isolated by Regulatory T Cell Isolation Kit II(Miltenyi Biotec, Germany) at the ratio of 1:0, 2:1 respectively and stimulated with human CD3/CD28 T cell activator (Stem Cell, Canada) at a dose of 25 µL/mL for 4 d in 37 °C, 5% CO_2_ incubator, and then analyzed by fluorescence-activated cell sorting (FACS).

### Flow cytometry

**Fig. 1:**
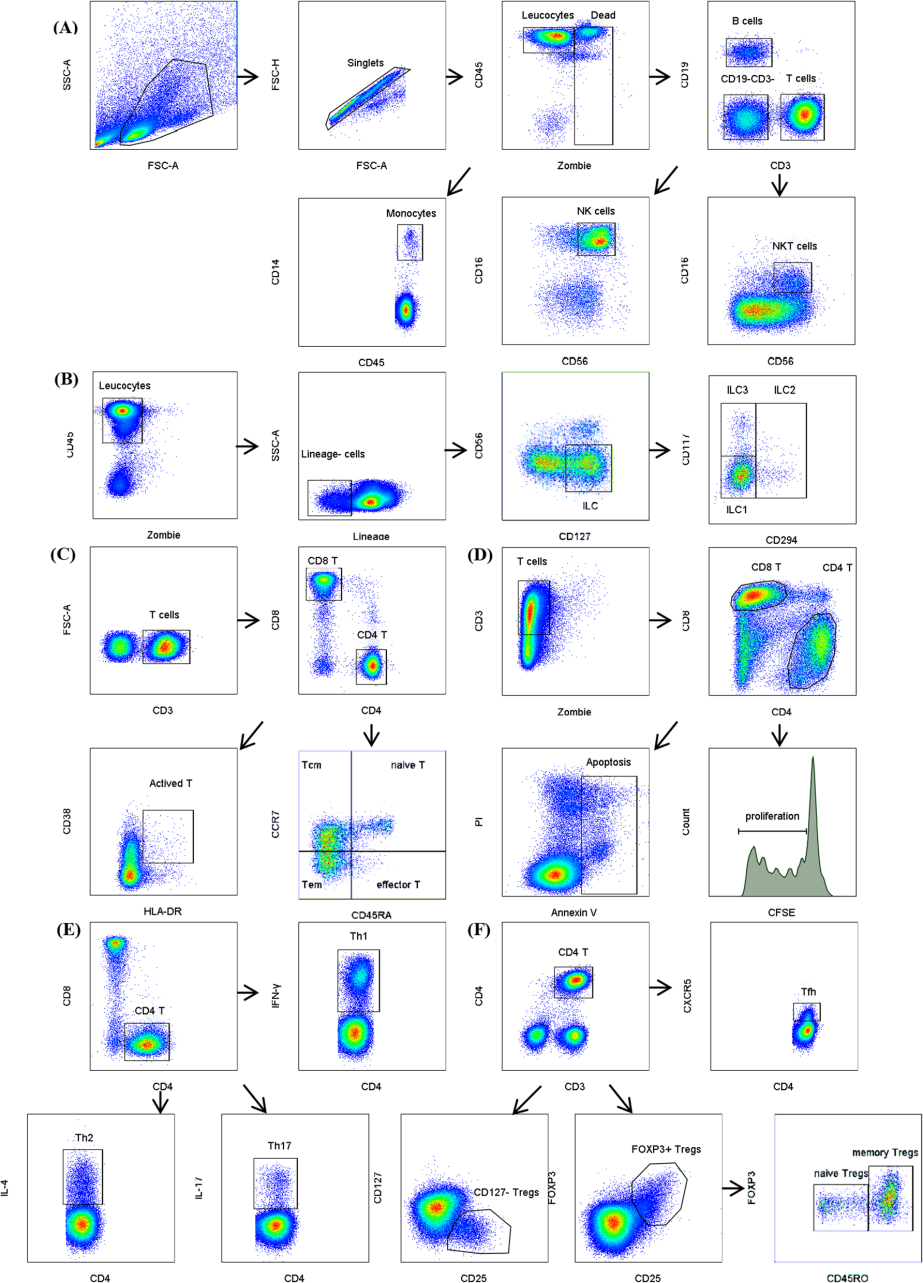
Gating strategies by flow cytometry. **A**, **B** Gating strategies for PBMCs subtypes. **C** Gating strategies for Th, Tc and Tcm, naive T, Tem, effector T. **D** Gating strategies for T cell apoptosis and proliferation. **E** Gating strategies for functional T cell subtypes-Th1, Th2, Th17. **F** Gating strategies for Tfh, Tregs and naïve Tregs,memory Tregs

The PBMCs were washed twice and resuspended in 100 μL of phosphate-buffered saline (PBS), and then stained with LIVE/DEAD Fixable Aqua Stain (Biolegend, USA) for the cell viability testing, and then incubated with surface markers in the dark for at least 30 min.Then, the PBMCs were fixed and permeabilized after surface marker staining and stained with intracellular cytokines such as IFN-r, IL-4, IL-17, or the transcription factor FOXP3. The cell gating strategies are shown in Fig. 1, and the color scheme and antibody information are shown in Additional file 1: Table S1. Data were acquired by BD Canto II Flow Cytometry and analyzed using the BD FACSDiva software or FlowJo.v10 software (BD, USA).

### Statistical analysis

The total cell numbers were calculated through multiple comparison analysis testing in two-way analysis of variance (ANOVA), and the other data were calculated using one-way ANOVA multiple comparisons, wherein *P* < 0.05 was considered statistically significant. Statistical analysis of the results was performed using GraphPad Prism 8.0 (GraphPad Software Inc., USA).

## Results

### PBMC recovery and viability remained stable after long-term cryopreservation

**Fig. 2:**
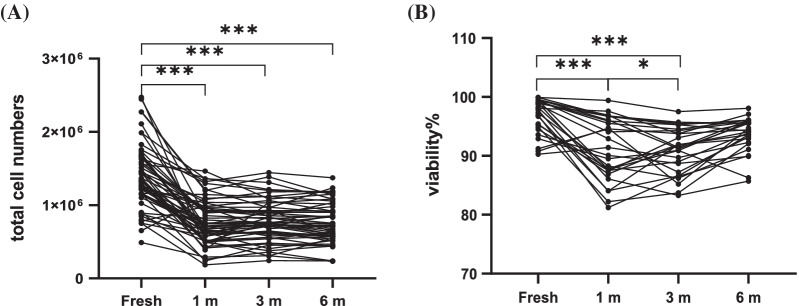
PBMCs recovery and viability. **A** total cells of PBMCs after cryopreservation(n = 57) and **B** proportions of live PBMCs (n = 20) in fresh isolated and cryopreserved (1, 3 and 6 mo, respectively) PBMCs

Fifty-seven peripheral blood samples were randomly obtained from the Xijing Hospital. After obtaining PBMCs by density gradient centrifugation, the cells of each patient were divided into five tubes, the fresh sample was used for subsequent experiments, and the remaining four samples were frozen for preservation to determine whether frozen time could affect the quality of PBMCs. The total cell numbers were detected using a Bio-Rad automatic cell counter, and the total number of frozen PBMCs was significantly reduced compared with that in fresh samples (P < 0.0001). However, there was no significant change after cryopreservation for 1, 3, or 6 months, and the statistical results showed 1 m versus 3 m, P = 0.48; 1 m versus 6 m, P = 0.84; 3 m versus 6 m, P = 0.11 (Fig. 2A). The viability of the PBMCs was also reduced significantly after cryopreservation (P < 0.0001) but remained stable during the different cryopreservation times, the statistical results showed that 1 m versus 3 m, P = 0.99; 1 m versus 6 m, P = 0.10; 3 m versus 6 m, P = 0.05 (Fig. 2B), indicating that although cryopreservation affects the cell recovery efficiency and viability of PBMCs, PBMCs still maintain a stable state during long-term cryopreservation.

### T cell subtypes in the pbmcs were not susceptible to long-term cryopreservation

**Fig. 3:**
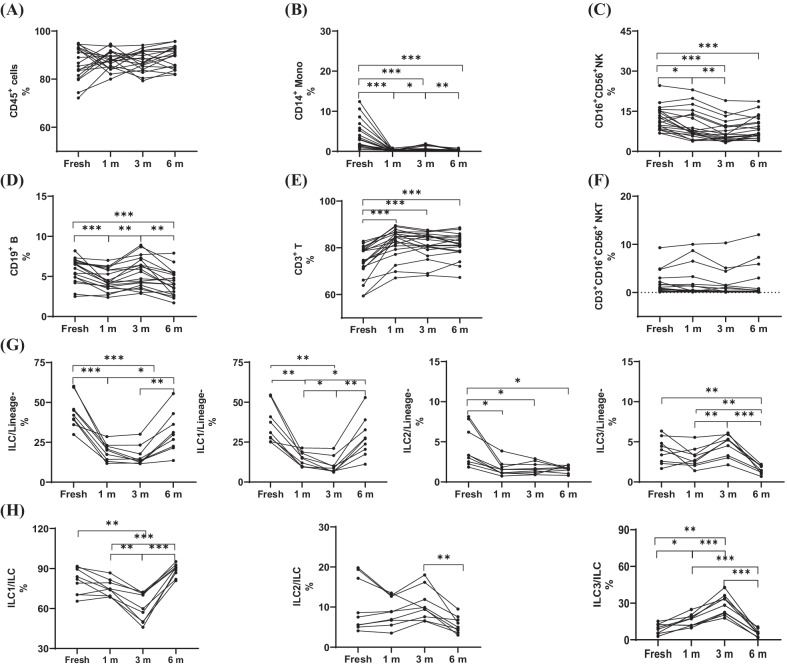
Effects of long-term cryopreservation on PBMCs subtypes. **A** The proportions of leucocytes. **B** The proportions of CD14^+^ Monocytes in CD45^+^ gate. **C** The proportions of CD16^+^CD56^+^ NK in CD45^+^ gate. **D** The proportions CD19^+^ B in CD45^+^ gate. **E** The proportions CD3^+^ T in CD45^+^ gate. **F** The proportions CD3^+^CD16^+^CD56^+^ NKT in CD45^+^ gate. The tested samples are fresh isolated and cryopreserved(1, 3 and 6 mo, respectively) PBMCs. **G** The proportions of total ILC and the subtypes ILC1,ILC2,ILC3 in Lineage^−^lymphocytes or **H** the proportions of the subtypes ILC1,ILC2,ILC3 in total ILC.*P < 0.05, **P < 0.01,***P < 0.001

Peripheral blood mononuclear cells contain two kinds of immune cell subtypes, namely, innate immune cells and adaptive immune cells. To further elucidate the changes in the PBMC subtypes after cryopreservation, the expression of the immune cell markers between freshly isolated and cryopreserved PBMCs were detected by flow cytometry. Firstly, the proportion of total leukocytes showed no significant difference during long-term cryopreservation. Then, the phenotypes of the innate immune cells were observed(Fig. 3A). Compared with the freshly isolated PBMCs, the number of monocytes and ILC were not only reduced significantly after cryopreservation (P < 0.01 for monocytes; P < 0.01 for ILC) but also changed dynamically during long-term cryopreservation (1 m vs. 3 m, 3 m vs. 6 m, P < 0.05, P < 0.05 for monocytes; 1 m vs. 6 m, 3 m vs. 6 m, P < 0.05, P < 0.01 for ILC) (Fig. 3B, G). Further analysis of the ILC subtypes showed that ILC1 and ILC3 were affected by long-term cryopreservation (1 m vs. 3 m, 1 m vs. 6 m, 3 m vs. 6 m, P < 0.05, P < 0.05, P < 0.01 for ILC1; 1 m vs. 3 m, 1 m vs. 6 m, 3 m vs. 6 m, P < 0.01, P < 0.01, P < 0.01 for ILC3), except for ILC2 (Fig. 3G), and the proportions of these subtypes in ILC were changed either during long-term cryopreservation or in freshly isolated PBMCs(Fig. 3H). Although the number of natural killer (NK) cells decreased significantly compared with that of the freshly isolated cells (Table 1), the NK cells remained stable during long-term cryopreservation (Fig. 3C). The phenotypes of the adaptive immune cells were observed, and there was a significant change in the percentages of T cells and B cells between the fresh and cryopreserved adaptive immune cells (Fig. 3D, Table 1). However, there was no difference in the percentages of T cells and natural killer T (NKT) cells between the PBMCs cryopreserved at different times (Fig. 3E, F).

**Table 1:** The effect of fresh isolation and cryopreservation on innate and adaptive immune cells

	Fresh (%)	1 m (%)		3 m (%)		6 m (%)	
	Mean (range)	Mean (range)	Fresh versus 1 m P	Mean (range)	Fresh versus 3 m P	Mean (range)	Fresh versus 6 m P
Lymphocytes	86.6 (72.2–94.9)	87.8 (80–94.6)	ns	87.5 (79.3–94.1)	ns	89.3 (81.9–95.7)	ns
T	73.9 (59.4–80.7)	82.2 (67.1–89.5)	< 0.0001	81.3 (68.2–87.6)	< 0.0001	81.0 (67.3–88.6)	< 0.0001
B	5.77 (2.5–7.3)	4.56 (2.4–7.0)	0.0004	5.51 (3.0–7.7)	ns	4.19 (1.7–7.9)	0.0004
NK	12.3 (6.8–24.6)	10.0 (4.1–23.0)	0.0136	7.6 (3.2–19.0)	< 0.0001	8.9 (3.9–18.7)	< 0.0001
NKT	1.7 (0.1–9.3)	1.8 (0.1–10)	ns	1.3 (0.1–10.3)	ns	1.6 (0.1–12)	ns
Monocyte	3.9 (0.5–12.4)	0.2 (0.0–0.8)	0.0004	0.6 (0.0–1.9)	0.0008	0.2 (0.0–0.8)	0.0004
ILC	44.1 (29.9–60.2)	19.1 (11.8–28.6)	0.0002	16.6 (11.6–30.1)	0.0004	31.1 (13.6–55.6)	ns
ILC1	36.0 (25.1–54.6)	14.4 (9.4–21.4)	0.0013	10.3 (6.2–21.1)	0.0012	28.0 (11.1–53.0)	ns
ILC2	4.3 (1.8–8.1)	1.7 (0.7–3.7)	0.0176	1.7 (0.9–2.9)	0.0148	1.6 (0.8–2.1)	0.0308
ILC3	3.9 (1.7–6.3)	3.0 (1.4–5.6)	ns	4.5 (2.1–6.1)	ns	1.5 (1.0–2.2)	0.0088

### T cell proportion, apoptosis, and proliferation were not affected by long-term cryopreservation

**Fig. 4:**
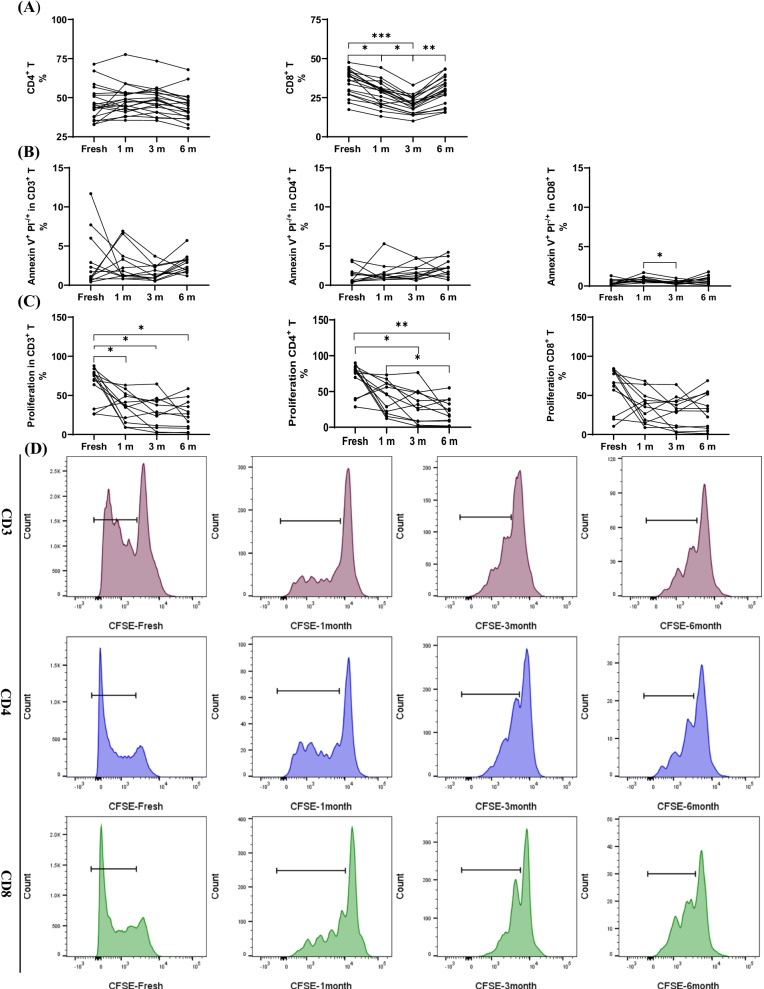
Effects of long-term cryopreservation on Tc, Th, apoptosis and proliferation (n = 20, 12, 12 respectively). **A** The proportion of Th and Tc in total T cells and **B** the proportion of apoptosis and **C** proliferation in fresh isolated and cryopreserved (1, 3 and 6 mo, respectively) PBMCs. **D** The proliferation results of flow cytometry in fresh isolated and cryopreserved (1, 3 and 6 mo, respectively) PBMCs.*P < 0.05, **P < 0.01,***P < 0.001

T cell response is an important part of cellular immunity and is involved in various types of biological functions against diseases and infections. Previous results have already confirmed the stability of T cell proportion after cryopreservation for 1, 3, and 6 months, respectively. T cells can be divided into CD4^+^ helper T cells and CD8^+^ cytotoxic T cells. The percentages of CD4^+^ T had no difference between the freshly isolated and cryopreserved PBMCs. Interestingly, the percentages of CD8^+^ T cells in the cryopreserved PBMCs were significantly reduced when compared with that in freshly isolated PBMCs (fresh vs. 1 m, 3 m, 6 m, P < 0.0001, P < 0.0001, P < 0.001; 1 m vs. 3 m, 6 m, P < 0.0001, P < 0.0001), indicating that the number of T cells decreased after cryopreservation which was mainly influenced by CD8^+^ T cells (Fig. 4A).

Although the viability of PBMCs have been proven to remain stable during cryopreservation in previous studies, cryopreservation may disrupt the integrity of the cell membrane, change the mitochondrial membrane potential, and cause cell apoptosis. The results indicated that the apoptosis of CD4^+^ T and CD8^+^ T cells were not affected by cryopreservation (Fig. 4B). On the other hand, proliferation of CFSE-labeled T cells was assessed, and the proliferation capacity between the freshly isolated and cryopreserved PBMCs changed significantly after stimulation with T cell activator and IL-2 (fresh vs. 1 m, 3 m, 6 m, P < 0.05, P < 0.05, P < 0.05), but no significant change was observed after prolonging the freezing time. This change in T cells was mainly caused by the CD4^+^ T cells, and the proliferation of CD8^+^ T cells was unchanged between the freshly isolated and cryopreserved PBMCs during cryopreservation (Fig. 4C). Although the proliferation ratio of the T cells was not affected by the extension of freezing time, it seemed that the passages of the T cells were changed, and after 72 h of stimulation under the same conditions, the number of proliferating cells cryopreserved for 3 or 6 months was significantly less than that of the cells cryopreserved for 1 month (Fig. 4D). In the experiment, we also found that sufficient cell numbers had a great influence on the results of proliferation (data not shown).

### Proportions of the activated T cells, naïve T cells, central memory T cells, effector T cells, and effector memory T cells were dynamically changed after long-term cryopreservation

**Fig. 5:**
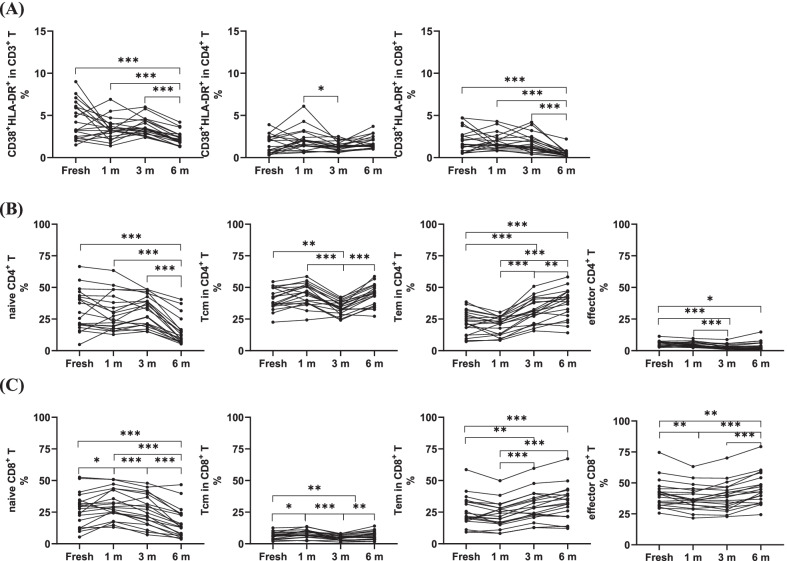
Effects of long-term cryopreservation on actived T cells and the T cell subtyes expressing CCR7 and CD45RA(n = 20). **A**The proportion of CD38^+^HLA-DR^+^ T cells,Th and Tc. **B **The proportion of Th Naïve(CD45RA^+^CCR7^+^)Tcm(CD45^−^CCR7^+^)Tem(CCR7^−^CD45RA^−^)effector T(CD45RA^+^CCR^−^). **C** The proportion of Tc Naïve(CD45RA^+^CCR7^+^)Tcm(CD45^−^CCR7^+^)Tem(CCR7^−^CD45RA^−^)effector T(CD45RA^+^CCR^−^) in fresh isolated and cryopreserved (1, 3 and 6 mo, respectively) PBMCs. *P < 0.05, **P < 0.01,***P < 0.001

Further study is necessary as there are several T cell subtypes according to their status and activation. Proportion of activated CD3^+^ T cells decreased significantly not only between the freshly isolated and cryopreserved PBMCs but also in the different cryopreservation times (fresh vs. 6 m, P < 0.01; 1 m vs. 3 m, 3 m vs. 6 m, P < 0.01, P < 0.0001). This change was mainly caused by CD8^+^ T cells (Fig. 5A, Table 2).

**Table 2:** Effect of long-term cryopreservation on different stage of T cells

	Fresh (%)	1 m (%)		3 m (%)			6 m (%)			
	Mean (range)	Mean (range)	Fresh versus 1 m P	Mean (range)	Fresh versus 3 m P	1 m versus 3 m P	Mean (range)	Fresh versus 6 m P	1 m versus 6 m P	3 m versus 6 m P
*CD4*										
Naïve	31.9 (4.9–66.5)	30.1 (12.8–63.4)	ns	31.6 (15.0–48.4)	ns	ns	14.8 (5.4–40.5)	< 0.0001	< 0.0001	< 0.0001
Central memory	41.0 (22.5–54.5)	44.3 (24.3–58.6)	ns	34.1 (24.1–42.3)	0.0013	< 0.0001	44.0 (27.2–58.7)	ns	ns	< 0.0001
Effector	4.8 (2.5–11.4)	4.8 (2.4–9.7)	ns	2.9 (0.9–8.8)	< 0.0001	< 0.0001	3.8 (0.8–14.8)	0.0264	ns	ns
Effector memory	22.4 (7.2–38.5)	20.2 (8.2–30.4)	ns	31.6 (15.7–50.0)	< 0.0001	< 0.0001	72.4 (14.1–58.3)	< 0.0001	< 0.0001	0.0014
Actived	1.5 (0.3–3.9)	2.1 (0.6–6.1)	ns	1.4 (0.6–2.5)	ns	0.0467	1.8 (1.0–3.7)	ns	ns	ns
*CD8*										
Naïve	27.1 (5.4–52.6)	31.3 (13.1–50.9)	0.0449	26.7 (7.1–47.9)	ns	0.0003	16.6 (3.7–46.7)	< 0.0001	< 0.0001	< 0.0001
Central memory	6.5 (1.9–12.5)	8.1 (2.3–13.6)	0.0178	4.7 (1.3–8.0)	0.0073	0.0001	6.2 (1.7–14.0)	ns	ns	0.0091
Effector	41.0 (25.6–74.6)	37.9 (21.6–63.2)	0.0065	39.1 (22.8–70.1)	ns	ns	45.1 (24.2–79.3)	0.0053	< 0.0001	< 0.0001
Effector memory	25.3 (25.6–58.2)	23.2 (21.6–63.2)	ns	29.5 (22.8–70.1)	0.0024	< 0.0001	32.1 (24.4–79.3)	< 0.0001	< 0.0001	ns
Actived	2.0 (0.5–4.7)	1.9 (0.8–4.3)	ns	1.7 (0.4–4.2)	ns	ns	0.5 (0.1–2.2)	< 0.0001	< 0.0001	< 0.0001

T cells, whether expressing C–C chemokine receptor type 7 (CCR7) and CD45RA or not, can be divided into naïve T cells, central memory T cells, effector T cells, and effector memory T cells, and can be further divided into CD4^+^ T or CD8^+^ T cells. Compared with that in freshly isolated cells, the proportions of naïve CD4^+^ T cells and naïve CD8^+^ T cells both decreased after cryopreservation for 6 months, and these changed dynamically at different freezing times. Although the proportion of CD4^+^ T central memory (Tcm) and CD8^+^ Tcm decreased significantly at 3 months, there was no significant change in 6 months as compared to that in freshly isolated cells. The proportions of effector memory cells (Tem) in both the CD4^+^ or CD8^+^ T cells, which decreased slightly after 1 month of cryopreservation, increased significantly with the extension of the freezing time (Fig. 5B, C, Table 2). The effector CD4^+^ and CD8^+^ T cells also tended to increase after a decrease in the cryopreservation time (Fig. 5B, C, Table 2). These results suggest that cryopreservation resulted in a significant reduction in the proportion of naïve T cells and that other subtype T cells increased after long-term cryopreservation but still differed from that in freshly isolated samples.

### Functional T cells remained stable after long-term cryopreservation, expect for tregs

**Fig. 6:**
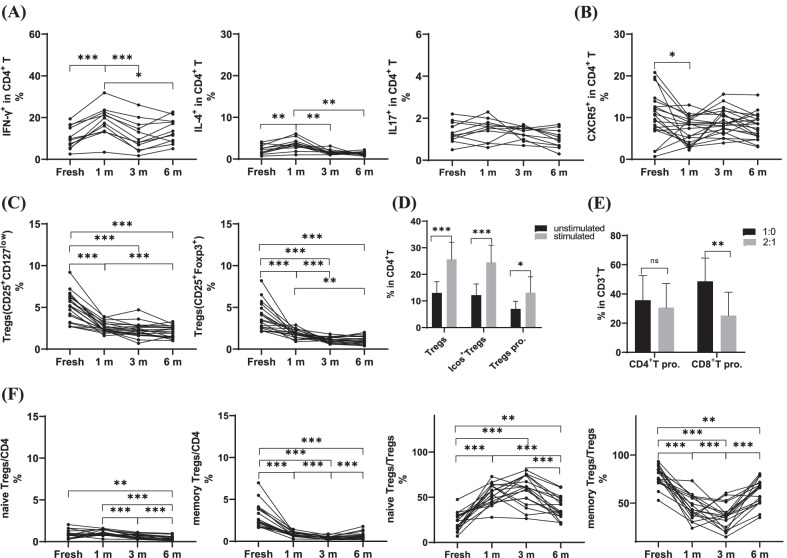
Effects of long-term cryopreservation on functional T cells-Th1, Th2, Th17(n = 12)and Tfh, Tregs(n = 18).The proportions of IFN-γ^+^Th1,IL-4^+^Th2,IL-17^+^Th17 (**A**) and CXCR5^+^Tfh (**B**) in fresh isolated and cryopreserved (1, 3 and 6 mo, respectively) PBMCs. **C **The proportions of CD4^+^CD25^+^Tregs expressing CD127^−^ or Foxp3^+^. **D** The frequency of Tregs, actived Tregs, proliferated Tregs after long-term cryopreservation. **E** Suppressive experiment using the CFSE label PBMCs cocultured with cryopreserved Tregs. **F** CD45RO^−^Naïve Tregs,CD45RO^+^Tregs in total CD4^+^T cells or in Tregs in fresh isolated and cryopreserved (1, 3 and 6 mo, respectively) PBMCs. *P < 0.05, **P < 0.01,***P < 0.001

To determine whether functional T cells were affected by long-term cryopreservation, the Th cells were marked with IFN-γ, IL-4, and IL-17, the T follicular helper cells (Tfh) were marked with CD45RO and C-X-C motif chemokine receptor 5 (CXCR5), and the Tregs were marked with CD25, CD127, and FOXP3. As expected, the functional T cells remained stable after long-term cryopreservation, and compared with that in freshly isolated PBMCs, the percentage of inflammatory T cells Th17 remained stable both before and after cryopreservation, Th1 and Th2 increased after 1 month of cryopreservation, and then remained stable after longer cryopreservation (Fig. 6A, Table 3). The number of Tfhs decreased slightly after cryopreservation for 1 month and remained unchanged after 3 months or longer (Fig. 6B, Table 3). The CD4^+^ T cells, which express the surface marker CD25 together with intracellular FOXP3, are Tregs. Down-modulated IL-7 receptor CD127 is also used to identify Tregs. Percentages of the CD25^+^CD127^low^ Tregs reduced significantly after cryopreservation, but there was no noticeable change after long-term cryopreservation. The results of CD25^+^FOXP3^+^Tregs led to the same conclusion (Fig. 6C, Table 3). Moreover, the function of cryopreserved Tregs were tested. It was showed that the frequency of Tregs, actived Tregs, proliferated Tregs increased significantly when 1 year cryopreserved PBMCs were induced into Tregs.The result of suppressive experiment also indicated that cryopreserved Tregs maintained suppressive function after long-term cryopreservation, especially the capacity of suppressing the proliferation of CD8^+^T. (Fig. 6D, E, Additional file 4: Fig. S3)The percentages of naïve Tregs and memory Tregs in the CD4^+^ T cells that were identified with CD45RO decreased significantly after cryopreservation and remained unchanged with increasing freezing time, and the proportions of naïve Tregs and memory Tregs in the Tregs dramatically changed (Fig. 6F, Table 3).

**Table 3:** Effect of long-term cryopreservation on functional T cells

	Fresh (%)	1 m (%)		3 m (%)			6 m (%)			
	Mean (range)	Mean (range)	Fresh versus 1 m P	Mean (range)	Fresh versus 3 m P	1 m versus 3 m P	Mean (range)	Fresh versus 6 m P	1 m versus 6 m P	3 m versus 6 m P
Th1	10.4 (5.4–19.4)	18.1 (3.4–31.9)	0.0003	11.1 (1.7–26.0)	ns	< 0.0001	13.7 (5.1–22.7)	ns	0.0293	ns
Th2	2.2 (0.7–4.1)	3.5 (1.0–6.1)	0.0048	1.6 (1.0–3.1)	ns	0.0057	1.3 (0.7–2.2)	ns	0.0010	ns
Th17	1.3 (0.5–2.2)	1.5 (0.6–2.3)	ns	1.3 (0.7–1.7)	ns	ns	1.0 (0.7–1.7)	ns	ns	ns
Tfh	10.4 (1.9–20.8)	7.1 (2.2–13.0)	0.0491	9.0 (5.0–15.6)	ns	ns	7.9 (3.0–15.4)	ns	ns	ns
Tregs(CD25^+^CD127^low^)	5.1 (2.7–9.2)	2.6 (1.6–3.9)	< 0.0001	2.3 (0.7–4.7)	< 0.0001	ns	2.1 (1.0–3.3)	< 0.0001	< 0.0001	ns
Tregs(CD25^+^Foxp3^+^)	3.8 (2.1–8.2)	1.8 (0.9–2.9)	0.0006	1.1 (0.6–1.8)	< 0.0001	0.0006	1.0 (0.4–2.0)	< 0.0001	0.0006	ns
naïve Tregs/CD4	0.9 (0.3–2.0)	1.0 (0.3–1.6)	ns	0.6 (0.3–1.1)	ns	0.0004	0.4 (0.1–1.0)	0.0026	< 0.0001	0.0003
memory Tregs/CD4	3.1 (1.7–7.0)	0.9 (0.3–1.4)	< 0.0001	0.4 (0.2–0.7)	< 0.0001	< 0.0001	0.7 (0.2–1.8)	< 0.0001	ns	0.0099

## Discussion

Peripheral blood mononuclear cells, which contain various immune cell types, play a crucial role in peripheral circulation; therefore, the quality of PBMCs needs to be controlled for clinical application or scientific research to obtain large numbers of samples to assess changes in the immune microenvironment during disease progression; that is, PBMCs should be cryopreserved for long periods of time [16]. Several reports have suggested that both isolation techniques and temperature fluctuations may affect cell viability and lymphocyte subtypes [17–19]. Cryopreservation is a relatively violent process in which freezing and thawing methods may cause physical and chemical stress on cells, resulting in changes in the cell surface markers [5]. In this study, autologous serum was used for cryopreservation, and cell numbers, viability, and subpopulations divided by different antibodies were assessed between freshly isolated and long-term cryopreserved PBMCs. Compared with freshly isolated PBMCs, both the number and viability of PBMCs were reduced after cryopreservation, but there was no significant difference during long-term cryopreservation; this result was consistent with the research that confirmed that PBMCs frozen from 2006 to 2015 maintained a high quality after recovery [15].

Peripheral blood mononuclear cells contain multiple immune cell types and perform different functions in the body. Although, in the research mentioned above, the PBMCs lasted a long period of time, the cryopreservation efficiency of the PBMC subtypes were not assessed further. For this purpose, we assessed innate immune cells and adaptive immune cells in PBMCs and concluded that the T, NK, and NKT cells did not change after long-term cryopreservation, while B cells, monocytes, and ILC changed significantly. In agreement with this, a previous study found that the number of B cells decreased and induced a higher total immunoglobulin (Ig) G production after 12 months of storage [20]. Although we confirmed that the proportion of T cells was not reduced during cryopreservation, the proportions of T cell subtypes and their status or functions may be affected by long-term cryopreservation, and the process of ice recrystallization or changes in osmotic pressure may damage the cytoskeleton and cell membrane [21]. The results of this study were similar to those of previous studies, which showed that T cells, especially CD4^+^ T cells, were not significantly affected by cryopreservation, regardless of cell proportion, active station, apoptosis, or proliferation [7, 22, 23].

Naive T cells, effector T cells, and Tem cells both in the CD4^+^ T and CD8^+^ T cells, which were not only compared with freshly isolated PBMCs but also in different cryopreserved times, were affected by cryopreservation. Some studies have reported that the number of Tcm increases and that of Tem decreases after 12 months of cryopreservation, and it could be concluded that these types of T cells seem to be more sensitive to the length of cryopreservation [7, 24, 25]. T follicular helper cells that express CXCR5 participate in information transmission or activation of B cells and maintain the humoral immune response for a long period of time [26]. Although our results showed significant changes in the B cells, Tfh was not affected by long-term cryopreservation after a slight decrease compared with fresh isolated PBMCs. Some studies have reported that the freeze–thaw process can result in a three to five-fold reduction of malaria antigen-specific IFN-γ-producing CD3^+^CD4^+^ effector populations, and others have suggested that cryopreservation in general leads to increased cytokine and chemokine responses, which is expected for IFN-γ in cultured PBMC supernatants [5, 27]. A recent report also suggested that there were no significant differences in the ratio of IFN-γ, IL-4, and IL-17 positive T cells between cell preparation tubes (CPT) and barrier-based tube option-Lymphoprep (LP) [28]. In our study, cytokines stimulated with phorbol 12-myristate 13-acetate remained stable after long-term cryopreservation, regardless of pro-inflammatory IFN-γ and IL-17 or anti-inflammatory IL-4. Several reports noted that cryopreservation was detrimental for the suppressive function and downregulated the key molecular features of Tregs; this was determined by single-cell RNA-sequencing [29, 30]. Our study found that both CD127^low^ Tregs and Foxp3^+^ Tregs were sensitive, and the ratio of cells rapidly decreased after initial cryopreservation. Then, the percentage of Tregs remained stable at a lower level after long-term cryopreservation, which agreed with the results of previous studies [31, 32]. Further studies confirmed that different states of Tregs, such as naïve Tregs and memory Tregs, significantly decreased in the CD4^+^ T cells and also dramatically changed in the Treg subpopulations after long-term cryopreservation. Although cryopreservation has a greater impact on Tregs, Tregs still maintain suppressive function after long-term cryopreservation. Notably, althougt Foxp3 is a highly specific marker for Treg cells, some effector CD4^+^ T cell can upregulate the expression of Foxp3 but have no regulatory/suppressive function[33]. The heterogeneity of human Foxp3+ regulatory T cells is an enssential and worthy issue in subsequent researches.

Finally, we truly considered the certain cell population loss may effect the frequency of other subsets during the experiment. So we counted absolute cell numbers and the results showed the similar trends to the frequency of cell subsets (Additional file 2: Fig. S1; Additional file 3: Fig. S2), which futher illustrated the reliability of the results in this manuscript.

## Conclusions

In the present study, we comprehensively evaluated the quality of long-term cryopreserved PBMCs and explored changes in the cell numbers, viability, and subtypes of PBMCs before and after cryopreservation. Particularly, changes in T cell apoptosis, proliferation, activation, and function were investigated and the effects of long-term cryopreservation on the same sample were analyzed. The results showed that long-term cryopreservation affected the activation of T cells and the function of Tregs, which are essential to the occurrence and development of immune diseases, and the imbalance of the ratio of effector T cells and Tregs will cause abnormal immune regulation in the body [34, 35]. Overall, the long-term cryopreservation of PBMCs can be partly used as a biological resource for clinical research or basic studies, but the effect of cryopreservation on PBMCs should be considered when selecting cell samples, especially in studying the function of activation or suppression. This study emphasized a comprehensive assessment of PBMC status after long-term cryopreservation without further exploring the effects of sample collection, including the concentration of cryopreserved cells, cryopreservation method (such as the concentration of cryoprotectant DMSO or fetal bovine serum), warming process, and even the use of anticoagulants. It is well known that the standardized management of PBMC biobanks is essential for the cell-based immune therapy in worldwide clinical trials; this study motivated us to pay more attention to the profound influence of cryopreservation itself on PBMCs in scientific research or clinical trials while improving the standard of biobank establishment.

## Supplementary Information

**Additional file 1.** **Table S1**: Antibody information

**Additional file 2.** **Figure S1**: The absolute cell count of long-term cryopreserved PBMCs subtypes. (A)leucocytes (B)CD14+ Monocytes in CD45+ gate (C)CD16+CD56+ NK in CD45+ gate (D)ILC in Lineage- lymphocytes (E)CD19+ B in CD45+ gate (F) CD3+ T in CD45+ gate (G)CD4+ Th (H) CD8+ Tc in fresh isolated and cryopreserved(1,3 and 6 mo,respectively) PBMCs.*P<0.05, **P<0.01,***P<0.001

**Additional file 3.** **Figure S2**: (A) The absolute cell count and proportions of ILC in cryopresered(6 and 12 month) PBMCs. *P<0.05, **P<0.01,***P<0.001

**Additional file 4.** **Figure S3**: Suppressive experiment using the CFSE label PBMCs cocultured with cryopreserved Tregs (A) The histogram results to show CD4-CFSE in the absence and presence of Tregs (B) The histogram results to show CD8-CFSE in the absence and presence of Tregs. The numbers represent the percentages of proliferative cells

## Data Availability

The datasets supporting the conclusions of this article are included within the article and its additional file.

## Abbreviations

PBMCs: Peripheral blood mononuclear cells
HSCs: Hematopoietic stem cells
NK: Natural killer cells
ILC: Innate lymphoid cells
Th: T helper cells
IFN-γ: Interferon-γ
IL-2: Interleukin-2
IL-4: Interleukin-4
IL-17: Interleukin-17
Tregs: Regulatory T cells
FOXP3: Intracellular forkhead box P3
GM-CSF: Granulocyte-macrophage colony-stimulating factor
TNF: Tumor necrosis factor
TGF-β: Transforming growth factor-β
FCM: Flow cytometry
HIV: Humanodeficiency virus
HLA: Human leukocyte antigen
EDTA: Ethylenediaminetetraacetic acid
DMSO: Dimethysulfoxide
CFSE: Carboxyfluorescein diacetate succinimidyl ester
FACS: Fluorescence-activated cell sorting
CCR7: C–C chemokine receptor type 7
Tcm: T central memory cells
Tem: T effector memory cells
CXCR5: C-X-C motif chemokine receptor 5
Tfh: T follicular helper cells
