# Supplementary material for: Comprehensive evaluation of the effects of long-term cryopreservation on peripheral blood mononuclear cells using flow cytometry
Source: BMC Immunol. 2022 Jun 7;23:30. doi: 10.1186/s12865-022-00505-4 (PMC9175382; doi:10.1186/s12865-022-00505-4)
Supplement: Supplementary file 1 — Additional file 1. Table S1: Antibody information [file 12865_2022_505_MOESM1_ESM.docx]

Supplemental table.1 Antibody information

|  | **Ab** | **Fluorochrome** | | **clone** | **Manufacturer** |
| --- | --- | --- | --- | --- | --- |
| **T,B,Monocyte,NK,NKT** | CD45 | | Percp cy5.5 | HI30 | Biolegend |
|  | CD3 | | PE-CY7 | UCHT1 | Biolegend |
|  | CD19 | | PE | HIB19 | Biolegend |
|  | CD56 | | APC-CY7 | HCD56 | Biolegend |
|  | CD14 | | APC | M5E2 | Biolegend |
|  | CD16 | | FITC | 3G8 | Biolegend |
|  | Zombie | | BV510 |  | Biolegend |
| **ILC** | CD45 | | PE-CY7 | HI30 | Biolegend |
|  | CD56 | | APC-CY7 | HCD56 | Biolegend |
|  | CD127 | | AF647 | A7R34 | Biolegend |
|  | CD117 | | PE | S18022G | Biolegend |
|  | CD294 | | BV421 | BM16 | Biolegend |
|  | Lineage | | FITC | cocktail | Biolegend |
|  | Zombie | | BV510 |  | Biolegend |
| **Th,Tc ,Tcm,naive T,Tem,effector T and activation** | CD3 | | PE-CY7 | UCHT1 | Biolegend |
|  | CD4 | | FITC | RPA-T4 | BD |
|  | CD8 | | APC | RPA-T8 | Biolegend |
|  | CD45RA | | Percp | HI100 | Biolegend |
|  | CCR7 | | BV510 | 3D12 | Biolegend |
|  | CD38 | | BV421 | HIT2 | Biolegend |
|  | HLA-DR | | APC-CY7 | L243 | Biolegend |
| **Apoptosis and proliferation** | CD3 | | PE-CY7 | UCHT1 | Biolegend |
|  | CD4 | | BV421 | RPA-T4 | BD |
|  | CD8 | | APC | RPA-T8 | Biolegend |
|  | FITC Annexin V Apoptosis Detection Kit I | | | | BD |
|  | CFSE Cell Division Tracker Kit Biolegend | | | | |
|  | Zombie | | BV510 |  | Biolegend |
| **Th1,Th2,Th17** | CD3 | | PE-CY7 | UCHT1 | Biolegend |
|  | CD4 | | FITC | RPA-T4 | BD |
|  | CD8 | | APC | RPA-T8 | Biolegend |
|  | IFN-γ | | Percp cy5.5 | B27 | Biolegend |
|  | IL-4 | | PE | 11B11 | Biolegend |
|  | IL-17 | | BV510 | TC11-18H10.1 | Biolegend |
|  | Zombie | | APC-CY7 |  | Biolegend |
| **Tregs,Tfh** | CD3 | | FITC | UCHT1 | Biolegend |
|  | CD4 | | BV421 | A161A1 | Biolegend |
|  | CD25 | | PE | BC96 | Biolegend |
|  | CD127 | | Percp cy5.5 | A019D5 | Biolegend |
|  | CD45RO | | PE-CY7 | UCHL1 | Biolegend |
|  | CXCR5 | | BV510 | J252D4 | Biolegend |
|  | Zombie | | APC-CY7 |  | Biolegend |
