# Supplementary material for: Comprehensive evaluation of the effects of long-term cryopreservation on peripheral blood mononuclear cells using flow cytometry
Source: BMC Immunol. 2022 Jun 7;23:30. doi: 10.1186/s12865-022-00505-4 (PMC9175382; doi:10.1186/s12865-022-00505-4)
Supplement: Supplementary file 2 — Additional file 2. Figure S1: The absolute cell count of long-term cryopreserved PBMCs subtypes. (A)leucocytes (B)CD14+ Monocytes in CD45+ gate (C)CD16+CD56+ NK in CD45+ gate (D)ILC in Lineage- lymphocytes (E)CD19+ B in CD45+ gate (F) CD3+ T in CD45+ gate (G)CD4+ Th (H) CD8+ Tc in fresh isolated and cryopreserved(1,3 and 6 mo,respectively) PBMCs.*P<0.05, **P<0.01,***P<0.001 [file 12865_2022_505_MOESM2_ESM.pdf]

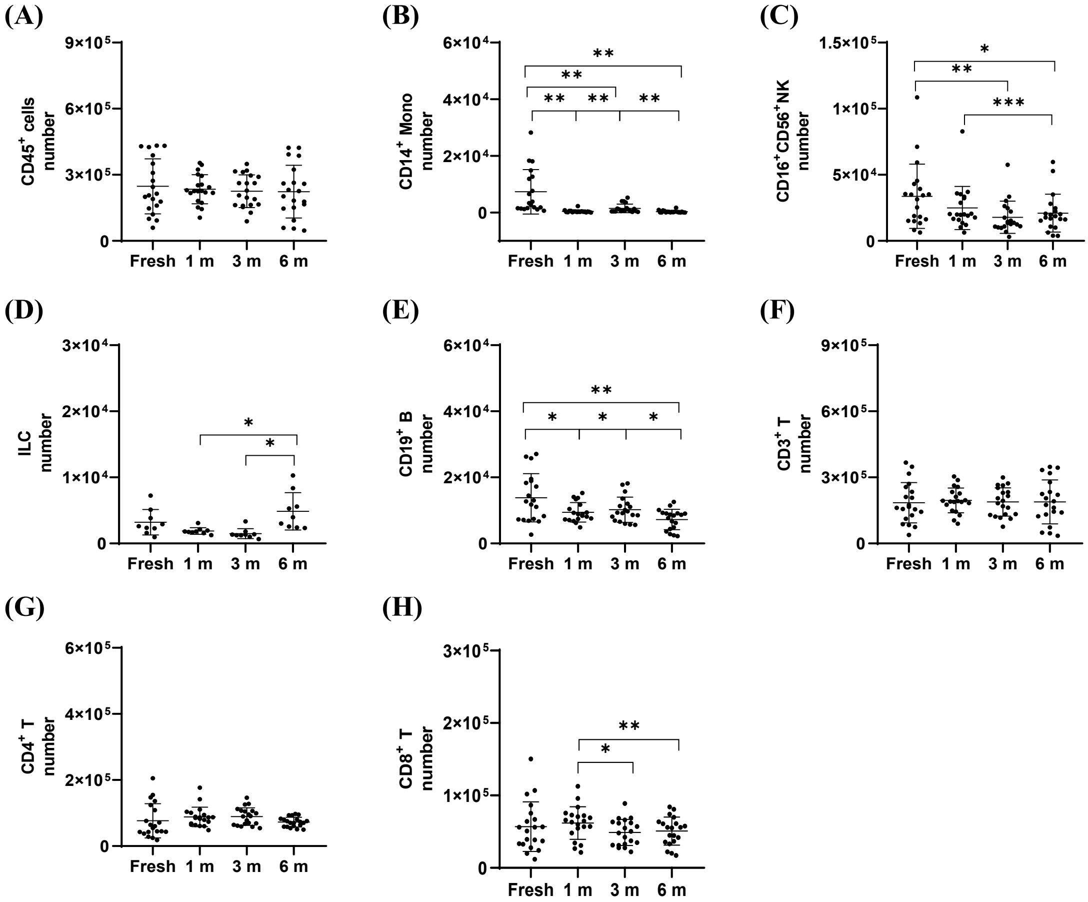

Supplemental Figure.1 The absolute cell count of long-term cryopreserved PBMCs subtypes. (A)leucocytes (B)CD14^{+} Monocytes (C)CD16^{+}CD56^{+} NK (D)ILC in Lineage^{-} lymphocytes (E)CD19^{+} B (F) CD3^{+} T (G)CD4^{+} Th (H) CD8^{+} Tc in fresh isolated and cryopreserved(1,3 and 6 mo,respectively) PBMCs.*P<0.05, **P<0.01,***P<0.001
