# Supplementary figures and images for: Comprehensive evaluation of the effects of long-term cryopreservation on peripheral blood mononuclear cells using flow cytometry
Source: BMC Immunol. 2022 Jun 7;23:30. doi: 10.1186/s12865-022-00505-4 (PMC9175382; doi:10.1186/s12865-022-00505-4)

(A)

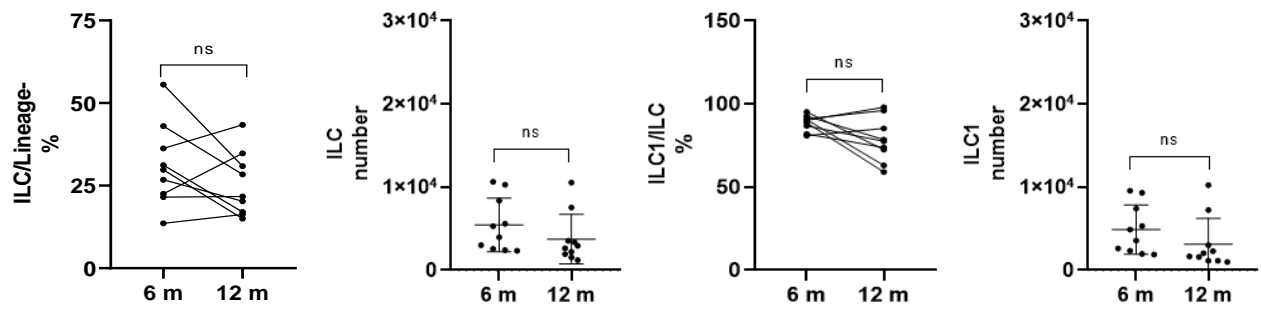

Supplement: Supplementary file 3 — Additional file 3. Figure S2: (A) The absolute cell count and proportions of ILC in cryopresered(6 and 12 month) PBMCs. *P<0.05, **P<0.01,***P<0.001 [file 12865_2022_505_MOESM3_ESM.pdf]

(A) PBMCs:Tregs  1:0  2:1

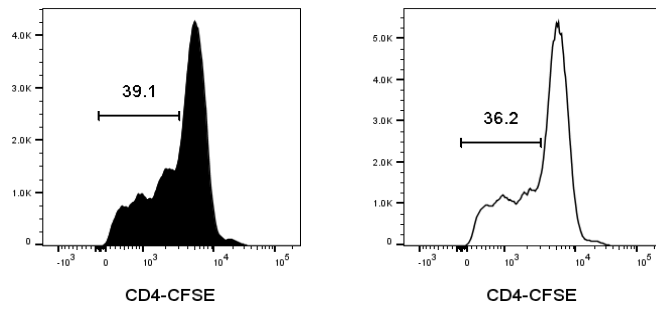

(B)

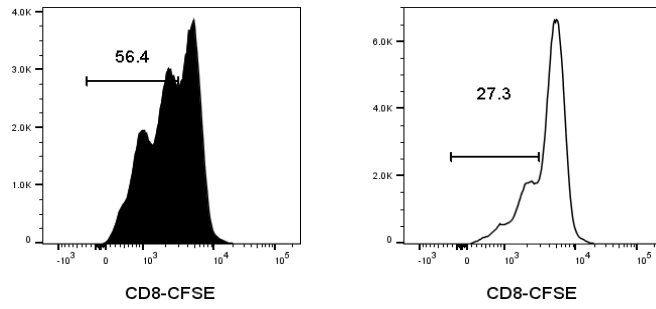

Supplement: Supplementary file 4 — Additional file 4. Figure S3: Suppressive experiment using the CFSE label PBMCs cocultured with cryopreserved Tregs (A) The histogram results to show CD4-CFSE in the absence and presence of Tregs (B) The histogram results to show CD8-CFSE in the absence and presence of Tregs. The numbers represent the percentages of proliferative cells [file 12865_2022_505_MOESM4_ESM.pdf]
